# TiO_2_ and Au-TiO_2_ Nanomaterials for Rapid Photocatalytic Degradation of Antibiotic Residues in Aquaculture Wastewater

**DOI:** 10.3390/ma12152434

**Published:** 2019-07-31

**Authors:** Tho Chau Minh Vinh Do, Duy Quoc Nguyen, Kien Trung Nguyen, Phuoc Huu Le

**Affiliations:** 1 Department of Drug Quality Control – Analytical Chemistry – Toxicology, Faculty of Pharmacy, Can Tho University of Medicine and Pharmacy, 179 Nguyen Van Cu Street, Can Tho City 94000, Vietnam; 2 Department of Physiology, Faculty of Medicine, Can Tho University of Medicine and Pharmacy, 179 Nguyen Van Cu Street, Can Tho City 94000, Vietnam; 3 Department of Physics and Biophysics, Faculty of Basic Sciences, Can Tho University of Medicine and Pharmacy, 179 Nguyen Van Cu Street, Can Tho City 94000, Vietnam

**Keywords:** TiO_2_ nanomaterials, Au nanoparticles, anodization, photocatalytic degradation of antibiotics, LC-MS/MS

## Abstract

Antibiotic residues in aquaculture wastewater are considered as an emerging environmental problem, as they are not efficiently removed in wastewater treatment plants. To address this issue, we fabricated TiO_2_ nanotube arrays (TNAs), TiO_2_ nanowires on nanotube arrays (TNWs/TNAs), Au nanoparticle (NP)-decorated-TNAs, and TNWs/TNAs, which were applied for assessing the photocatalytic degradation of eight antibiotics, simultaneously. The TNAs and TNWs/TNAs were synthesized by anodization using an aqueous NH_4_F/ethylene glycol solution. Au NPs were synthesized by chemical reduction method, and used to decorate on TNAs and TNWs/TNAs. All the TiO_2_ nanostructures exhibited anatase phase and well-defined morphology. The photocatalytic performance of TNAs, TNWs/TNAs, Au-TNAs and Au-TNWs/TNAs was studied by monitoring the degradation of amoxicillin, ampicillin, doxycycline, oxytetracycline, lincomycin, vancomycin, sulfamethazine, and sulfamethoxazole under ultraviolet (UV)-visible (VIS), or VIS illumination by LC-MS/MS method. All the four kinds of nanomaterials degraded the antibiotics effectively and rapidly, in which most antibiotics were removed completely after 20 min treatment. The Au-TNWs/TNAs exhibited the highest photocatalytic activity in degradation of the eight antibiotics. For example, reaction rate constants of Au-TNWs/TNAs for degradation of lincomycin reached 0.26 min^−1^ and 0.096 min^−1^ under UV-VIS and VIS irradiation, respectively; and they were even higher for the other antibiotics. The excellent photocatalytic activity of Au-TNWs/TNAs was attributed to the synergistic effects of: (1) The larger surface area of TNWs/TNAs as compared to TNAs, and (2) surface plasmonic effect in Au NPs to enhance the visible light harvesting.

## 1. Introduction

Titanium dioxide (TiO_2_) is one of the most widely studied materials for applications in solar cells [1,2,3], pollutant degradation [4,5,6], photolysis of water [7], gas sensing [8], and bio-applications [9,10], due to its excellent photocatalytic reactivity, high chemical stability, non-toxicity, biocompatibility, and low cost [11,12,13]. However, the large band gap of TiO_2_ (3.2 eV) limits it light absorption to only 5% of the solar spectrum [14,15,16]. Considerable effort has been made to improve the light absorption of TiO_2_ by doping with non-metals (N, F, S) [17,18,19] or chemical modification to narrow the band gap [20]. In addition, visible-light absorption can also be achieved by coupling TiO_2_ to small-band-gap quantum dots [21]. Recently, a new approach involving metal nanostructures in enhancing the visible-light photoactivity of TiO_2_ via plasmonic effect has received much attention [22,23,24,25,26]. Moreover, metal nanoparticles (NPs) have demonstrated good photo-stability [14]. 

TiO_2_ nanomaterials are of great interest because of their large surface area and high light absorption capability [27,28,29,30,31]. In this study, TiO_2_ nanotube arrays (TNAs) and TiO_2_ nanowires on nanotube arrays (TNWs/TNAs) are of interest, because they can provide a large surface-to-volume ratio and unidirectional electrical channel [32,33]. TNWs/TNAs presented a better photocatalytic degradation of methylene blue than that of TNAs, which was attributed to the presence of partial coverage of TNWs on the surface of TNAs for the enhanced surface area [6]. By using the anodic oxidation, the nanostructures of TNAs and TNWs/TNAs can be fabricated on immobilized Titanium folds that allows retrieval of the photocatalysts from the reaction solution after treatment, so they can be reused for many times. 

The aquaculture production sector of the Mekong Delta (Vietnam) has reached an annual production of 1.14 million tons in 2012 [34,35]. Antibiotics are commonly used in aquaculture for the prevention and treatment of diseases. However, Vietnam has very little enforced regulation pertaining to antibiotic usage in domestic aquaculture. Consequently, antibiotic residues in aquaculture wastewater of the Mekong Delta region are considered as an emerging environmental problem, due to their adverse effects on ecosystems, the aquaculture production and its economy [36,37], and human health [38,39,40,41,42]. Indeed, antibiotic residues in the environment have been found at low levels, usually in the ng·L^−1^–μg·L^−1^ range [38,39,40]. The antibiotic residues can result in bacterial antibiotic resistance [41,42], which in turn can be a serious risk to humans and other animals [37]. To address this environmental issue, photocatalysis has received tremendous attention, owing to its great potential in removing antibiotics from aqueous solutions via a green, economic, and effective process [43,44]. Indeed, photocatalytic degradation of tetracycline using nanosized titanium dioxide in an aqueous solution has been studied. Also, the degradation of paracetamol in aqueous solutions by TiO_2_ photocatalysis in powder and immobilized forms have been studied [45]. Y. He et al. studied the degradation of pharmaceuticals (i.e., Propranolol, Diclofenac, Carbamazepine, and Ibuprofen) in wastewater using immobilized TiO_2_ photocatalysis under simulated solar irradiation [46]. Therefore, the hypothesis of this study is that the antibiotic residues in aquaculture wastewater can be degraded effectively and rapidly by using nanostructured TiO_2_ and Au-TiO_2_ photocatalysts. 

In this study, we fabricated TNAs, TNWs/TNAs, Au NP-decorated TNAs, and Au NP- decorated TNWs/TNAs and utilized them to degrade antibiotic residues in aquaculture wastewater of the Mekong Delta (Vietnam) via the photocatalysis process. Indeed, for the first time, we successfully developed a sensitive, specified and repeatable analytic procedure to assess the photocatalytic removal efficiency of important classes of antibiotics, including amoxicillin (AMOX), ampicillin (AMPI), doxycycline (DXC), oxytetracycline (OTC), lincomycin (LCM), vancomycin (VCM), sulfamethazine (SMT), and sulfamethoxazole (SMZ) simultaneously in aquaculture wastewater, using liquid chromatography/tandem mass spectrometry (LC-MS/MS) analysis. LC-MS/MS is the combination of liquid chromatography (LC) with mass spectrometry (MS). Structural-morphological, and photocatalytic degradation kinetics of the eight antibiotics under UV and/or VIS irradiation are discussed in detail.

## 2. Experimental Details

TNAs and TNWs/TNAs were grown on Titanium (Ti) foil substrates (99.9% purity, 1 cm × 2.5 cm size, 0.4 mm thickness) by anodic oxidation. Prior to anodization, the substrate was first ultrasonically cleaned using acetone, methanol, and deionized water, followed by drying in a N_2_ gas flow. The anodization was performed using a two-electrode system with the Ti foil as an anode and a stainless steel foil (SS304) as a cathode. The electrolyte contained 0.3 wt % NH_4_F (SHOWA, Tokyo, Japan) in ethylene glycol (EG) solution with 2 vol % water. The Ti foil was anodized at 30 V for 1 h and 5 h to grow TNAs and TNWs/TNAs, respectively. The samples were then annealed at 400 °C for 1 h to induce sample crystallization. Au nanoparticles were prepared by chemical reduction method in which water (100 mL) containing HAuCl_4_. 4H_2_O (0.2 mM) and citric acid (0.5 mM) was stirred at 120 °C. The Au-TNAs and Au-TNWs/TNAs were prepared by immersing the samples in the Au solution for 6 h at room temperature. The samples were then annealed at 400 °C for 1h to improve the crystallinity and Au-TiO_2_ interfaces. 

The crystal structures of the nanomaterials were characterized by X-ray diffraction (XRD, Bruker D2, Bruker, Billerica, MA, USA) using Cu Kα radiation (λ = 1.5406 Å). Morphologies of the samples were characterized by scanning electron microscopy (SEM, JEOL JSM-6500, Pleasanton, CA, USA). An antibiotic solution was designed and prepared to reflect the practical aquaculture wastewater samples, collected at Dam Doi district of Ca Mau province, which is one of the large aquaculture areas of Mekong Delta, Vietnam. The aquaculture wastewater had a biochemical oxygen demand (BOD) of 10.7 mg/L, chemical oxygen demand (COD) of 19.6 mg/L, and low concentration of organic matter. The spiked mixture solution of standard eight antibiotics with an initial concentration of 500 ng/mL was dissolved in blank wastewater samples containing 0.1% (v/v) formic acid. Photocatalytic reactions were carried out by immersing a sample into a 30 ml antibiotic solution under UV-VIS at approximately 120 mW·cm^−2^ or VIS illumination at approximately 95mW·cm^−2^ using a 100 W Xenon lamp. Prior to illumination, the catalyst was immersed into the solution and magnetic stirring followed for 20 min in the dark, to ensure absorption‒desorption equilibrium between the photocatalyst (sample) and antibiotic solution. A band-pass filter for λ ≥ 400 nm was used to select the VIS spectrum region from the Xenon lamp. The reaction temperature was kept at 32–33 °C for all photocatalytic reactions. After a certain photocatalytic reaction time, qualitative and quantitative analysis of antibiotics was determined by LC-MS/MS technique. We used ultra performance liquid chromatography (Acquity H-Class, Waters, Milford, MA, USA) coupled with a triple quadrupole mass detector (Xevo-TQD, Waters, Milford, MA, USA), and equipped with an electrospray ionization (ESI) interface. Mass analysis was in positive and multiple-reaction monitoring (MRM) and daughter ion mode. The Agilent Poroshell 120 Phenyl-hexyl (4.6 × 150 mm; 2.7 µm) column was used, and the mobile phase included acetonitrile-methanol-aqueous formic acid 0.1% in gradient program [47]. The results were evaluated using the degradation percentage of each antibiotic at various reaction times, starting at 0 and followed by 2, 5, 9, 14, and 20 min, as the ratio between the initial peak area of antibiotic solution (without photocatalytic treatment) and peak area of treated antibiotic solution. It was possible to follow the degradation progress of every antibiotic by calculating these areas with Masslynx Software 4.1. 

## 3. Results and Discussion

**Figure 1:**
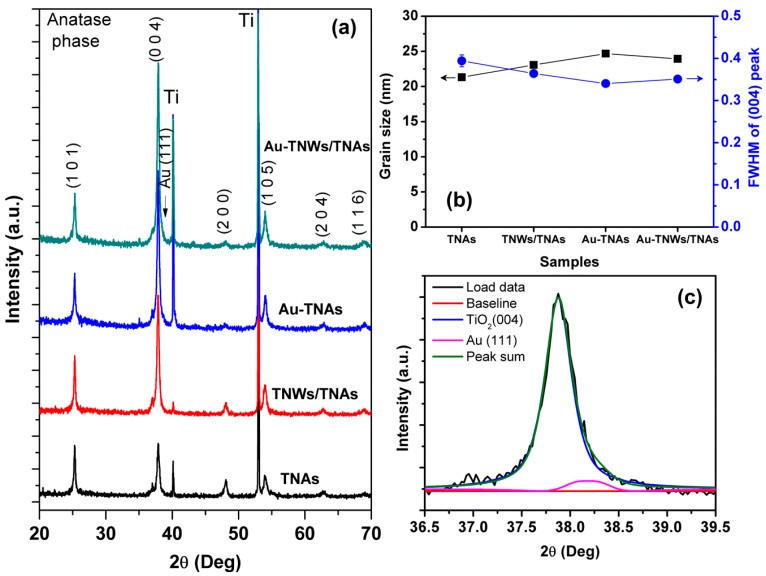
(**a**) The XRD patterns of TiO_2_ nanotube arrays (TNAs), TiO_2_ nanowires on nanotube arrays (TNWs/TNAs), Au-TNAs, and Au-TNWs/TNAs. (**b**) Grain size and the full width at half maximum (FWHM) of (004) peaks of the four nanomaterials. (**c**) The (004) peak of Au-TNAs shows two components of TiO_2_ (004) and Au (111).

Figure 1 shows the XRD patterns of TNAs, TNWs/TNAs, Au-TNAs, and Au-TNWs/TNAs. All the samples exhibited the anatase phase of TiO_2_ with preferred orientations of (004), (101) and (105) lattice planes at 37.8°, 25.1°, and 53.8°, respectively (JCPDS No. 21–1272). Also, there were no rutile peaks, indicating that the TiO_2_ nanomaterials in this study possessed a pure anatase phase. This result agreed with those reported in [4,5,13,19,48,49]. A closer inspection of the (004) peaks revealed that Au (111) component was found in the (004) peaks of Au-TNAs and Au-TNWs/TNAs, as demonstrated in Figure 1c, confirming the presence of crystalline Au NPs in these samples. 

The grain sizes (*D*) of the samples were estimated by using the Scherrer equation: *D* = 0.9*λ/β*cos*θ*, where *λ*, *β*, and *θ* are the X-ray wavelength, full width at half maximum of the anatase phase TiO_2_ (004)-oriented peak, and Bragg diffraction angle, respectively [50]. Clearly, the estimated grain size varied in a narrow range between 21.3 nm and 24.7 nm, and the full width at half maximum (FWHM) of the (004) peak remained almost constant (Figure 1b). Those results confirmed that the grain size and the crystallinity of four nanomaterials were almost the same. 

**Figure 2:**
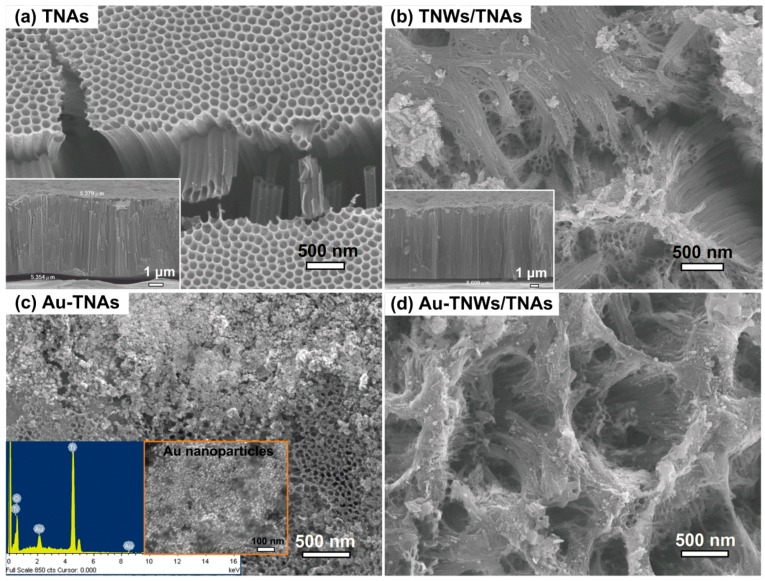
SEM images of (**a**) TNAs, (**b**) TNWs/TNAs, (**c**) Au-TNAs, and (**d**) Au-TNWs/TNAs. The insets in (**c**) show a typical EDS spectrum for Au-TNAs and Au-TNWs/TNAs, and the morphology of as-synthesized Au nanoparticles.

Figure 2 shows the morphology of TNAs, TNWs/TNAs, Au-TNAs, and Au-TNWs/TNAs. Clearly, the TNAs exhibited a highly ordered, uniformed, and clean surface. The TNAs had tube diameter of 75 nm and thickness of 5.4 µm (Figure 2a inset). In Figure 2b, TNWs/TNAs exhibited a TNWs (length of 6 µm) covering on the TNAs. The thickness of TNWs/TNAs film was 8.6 µm, as shown in the inset of Figure 2b. The inset in Figure 2c shows the morphology of as-synthesized Au nanoparticles with size of 20 ± 10 nm. For Au-TNAs samples, Au nanoparticles distributed relatively uniformly on the surface of TNAs (Figure 2c). In addition, a typical energy-dispersive X-ray spectroscopy (EDS) spectrum of Au-decorated TiO_2_ samples in this study is shown in the inset of Figure 2c. Obviously, Ti, O, Au peaks were observed, confirming the successful fabrications for Au-TNAs and Au-TNWs/TNAs samples. Finally, the morphology of Au-TNWs/TNAs can be observed in Figure 2d. 

During the anodization process, TNA growth is driven by the anodic-oxidation reaction (to form TiO_2_ from Ti) and the chemical dissolution of the TiO_2_ layer under the presence of electric field [19,51,52,53]. The reactions are given below: 

Anodic reaction: Ti + 2H_2_O − 4e → TiO_2_ + 4H^+^

Cathodic reaction: 4H^+^ + 4e → 2H_2_

Chemical etching (dissolution) reaction: TiO_2_ + 6F^−^ + 4H_4_^+^ → TiF_6_^2−^ + 2H_2_O

**Figure 3:**
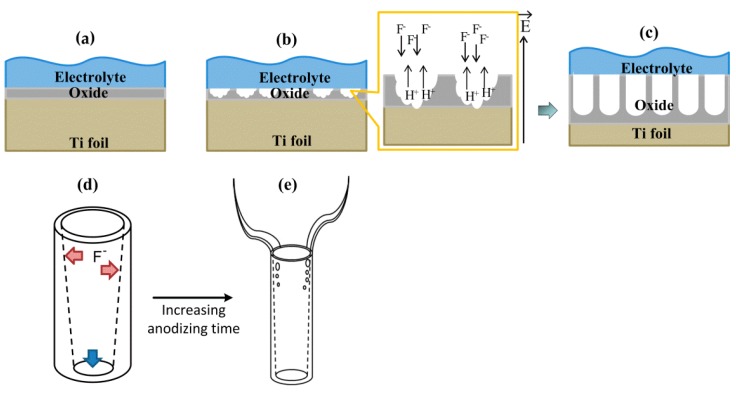
The growth process of TiO_2_ nanotube arrays (TNAs): (**a**) non-conductive thin oxide layer forming, (**b**) local growth of the pits, (**c**) growth of the semicircle pores and developed nanotube arrays, (**d**) The shape and wall thickness profile of TNAs prior to the emergence of nanowires (TNWs), (**e**) Schematic of the TNWs/TNAs structure.

The current density (*j*) changes with anodizing time (*t*) in an anodic oxidation process [53,54]. Initially, the *j* rapidly decreases, then slightly increases, and finally remains a constant [54]. According to the *j*–*t* characteristics, the TNAs growth process can be divided into three stages. In the early stage, the formation of a non-conductive thin oxide layer, associated with the decrease of *j* (Figure 3a). Next, there is the local growth of pits as evidenced by the slight increase of *j* (Figure 3b). Finally, the nanotube arrays are grown from the initial pits when *j* remains a constant (Figure 3c). When the dissolution rate of the wall of the nanopores is slower than that of the growth rate of nanopores, the diameter and length of the nanotubes will gradually increase. And, these sizes will remain unchanged when the growth rate is equal to the dissolution rate [53,55].

In the EG/H_2_O solution containing NH_4_F electrolyte, the migration of F^−^ toward the electric field at the bottom electrode is inhibited by the highly viscous solution. Thus, the F^−^ concentration at the tube mouth is much higher than it is at the tube bottom [6], while the chemical dissolution reaction is enhanced under the presence of H^+^ ions from water. Consequently, the tube wall thickness near the tube mouth was thinner than the lower sections, as shown in Figure 3d. By increasing anodizing time, strings of through holes are formed on the tube wall and they would initiate and propagate downward from the top to the bottom of TNAs (or along the F^−^ migration direction). Meanwhile, the holes near the top expand and connect to each other, and finally split into nanowires (Figure 3e) [6].

**Figure 4:**
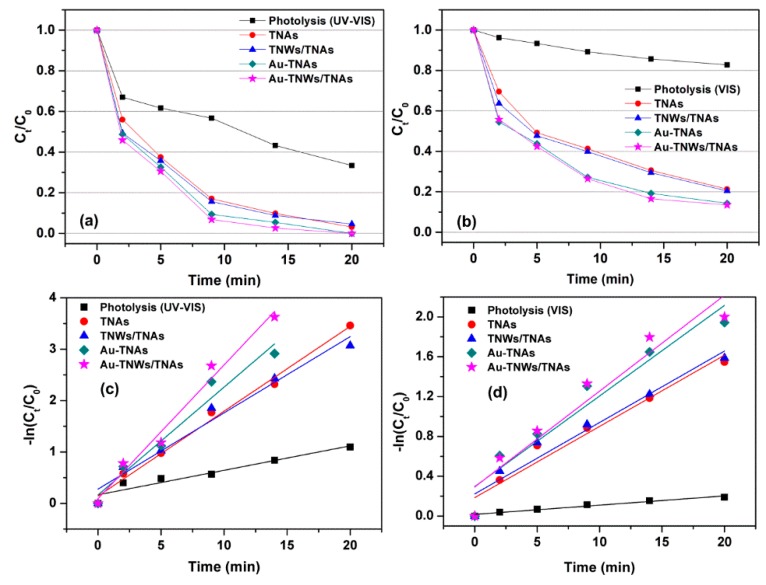
(**a**) Photocatalytic degradation of lincomycin (LCM, 500 ng/mL) using five reaction conditions of photolysis (UV-VIS), and photocatalysis (with TNAs, TNWs/TNAs, Au-TNAs, and Au-TNWs/TNAs). (**b**) Photocatalytic degradation of LCM under photolysis of the visible light (λ ≥ 400 nm of Xenon lamp) and the photocatalysis conditions. (**c**,**d**) LCM degradation kinetic curves of the five reaction conditions under UV-VIS illumination (**c**) and VIS illumination (**d**).

**Figure 5:**
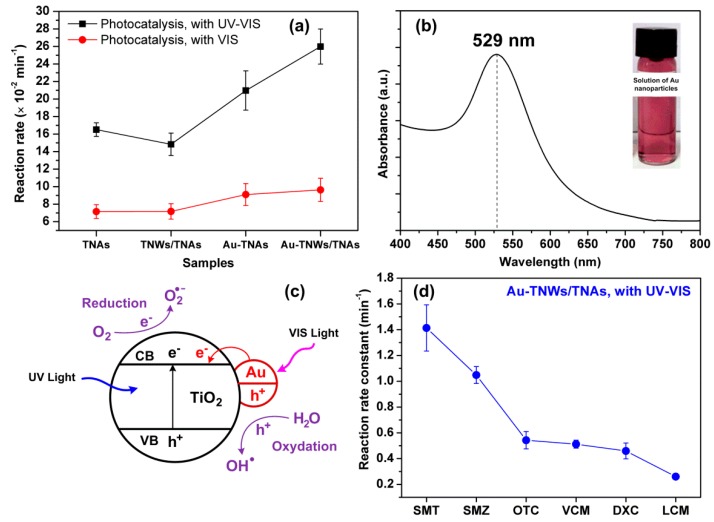
(**a**) Reaction rate constant (*k*) of various nanomaterials in photocatalytic degradation of Lincomycin (500 ng/mL) under UV-VIS and VIS irradiation. (**b**) Absorption spectrum of the solution of Au nanoparticles, showing the LSPR peak at 529 nm; and the inset image is a photograph of the Au nanoparticle solution. (**c**) A proposed mechanism for the photocatalytic activity of Au-TiO_2_ upon the excitation of the Au surface plasmon band. (**d**) Reaction rate constant of various antibiotics under photocatalysis using Au-TNWs/TNAs under UV-VIS irradiation. The antibiotic abbreviations: SMT, sulfamethazine; VCM, vancomycin; OTC, oxytetracycline; SMZ, sulfamethoxazole; DXC, doxycycline; LCM, lincomycin.

The photocatalytic degradation kinetic of LCM is used to evaluate the photocatalytic performance of the four nanomaterials. The pseudo-first-order rate constants were determined by fitting the data with the Langmuir–Hinshelwood kinetics rate model [56,57]. Figure 4a,b shows photocatalytic degradation of LCM using five reaction conditions, namely photolysis (UV-VIS or VIS), and photocatalysis with TNAs, TNWs/TNAs, Au-TNAs, and Au-TNWs/TNAs nanomaterials. Both photolysis and photocatalysis reactions generally follow the exponential decay, C_t_ = C_0_ × e^−^*^k^*^t^, where C_t_ is the concentration of antibiotic at time t (ng/mL), C_0_ is the initial concentration (ng/mL), and *k* is the reaction rate constant (min^−1^). By performing the linear fitting on the plot of –ln(C_t_/C_0_) versus reaction time t, the *k* is yielded, and the fittings are shown in Figure 4c,d. Specifically, the *k* values of LCM were 4.8 × 10^−2^ min^−1^ and 0.93 × 10^−2^ min^−1^ under UV-VIS and VIS irradiation, respectively. This indicates that UV irradiation degrades the antibiotics better than VIS, due to the higher photon energy via the photolysis effect [46,58,59]. As shown in Figure 4a,b, the photocatalysis shows significantly better performance in eliminating LCM than photolysis. The *k* values for LCM were in ranges of 14.8 × 10^−2^–26 × 10^−2^ min^−1^ under UV-VIS illumination and 7.2 × 10^−2^–9.5 × 10^−2^ min^−1^ under VIS illumination (Figure 5a). That means that the reaction rates of photocatalysis were 3.1–5.5 times and 7.6–10.3 times higher than those of UV-VIS photolysis and VIS photolysis, respectively.

Figure 5a shows the *k* values of the four kinds of nanomaterials under UV-VIS and VIS irradiation. Generally, the *k* of TNWs/TNAs is higher than that of TNAs, which is primarily attributed to the presence of partial coverage of TNWs on the surface of TNAs for the enhanced surface area [6,53]. There was a significant enhancement in the *k* values by decorating TNAs and TNWs/TNAs with Au NPs, because of the enhancement of the visible-light photoactivity of TiO_2_ via the localized surface plasmon resonance (LSPR) effect [14,22,60] (Figure 5a). The LSPR of spherical Au NPs (20 ± 10 nm diameter) in this study was suggested by the absorption peak at 529 nm (Figure 5b), which was well consistent with the LSPR-peaks of Au nanoparticles in [61,62]. In addition, the absorption enhancement in VIS region for Au-TiO_2_ was confirmed by the UV-VIS absorption spectra in [61,63]. LSPR can be described as the local electromagnetic fields near the surface of Au NPs being strongly enhanced when the electromagnetic field of the incident light becomes associated with the oscillations of the conduction electrons of Au NPs. Indeed, optical simulations clearly presented LSPR-enhanced electric fields at the interface of Au-TiO_2_, owing to photo-excited Au nanoparticles [64]. Herein, a proposed mechanism for enhanced photocatalytic activity of Au-TiO_2_ is that the LSPR-absorption of Au NPs generate photoexcited electrons and holes under VIS irradiation, and then the energetic electrons can inject into the conduction band of TiO_2_ and trigger photocatalytic reactions (Figure 5c) [61,62,65,66]. Therefore, Au-TNWs/TNAs possessed the highest photocatalytic performance amongst the four kinds of nanomaterials, due to the synergistic effects of large surface area and the LSPR effect.

Figure 5d summarizes the *k* values of various antibiotics treated using photocatalytic reaction of the Au-TNWs/TNAs (the best nanomaterial in this study) under UV-VIS irradiation. Here, the *k* is determined by the intrinsic photocatalytic property of the nanomaterial and the photolysis of antibiotics. AMOX and AMPI with β lactam ring structures decomposed rapidly by photolysis reaction with UV-VIS illumination [67]. In addition, the reaction rate of SMT and SMZ reached high values of 1.41 min^−1^ and 1.05 min^−1^, respectively; meanwhile, it was only 0.26 min^−1^ for LCM. That is because the former has amine bond structure [68], while LCM has amide bond structure [68]. Similarly, all the molecule structures with amide bonds of VCM, DXC, and OTC are more resistant to photolysis. Consequently, VCM, DXC, and OTC exhibited lower *k* values (1.05, 0.46, and 0.54 min^−1^) and needed a reaction time above 20 min to completely degrade. For comparison, the photocatalytic degradation rate of OTC using the Au-TNWs/TNAs (i.e., 0.54 min^−1^) was far higher the *k* of 0.032 min^−1^ using TiO_2_ nanobelts loading Au NPs [63].

**Figure 6:**
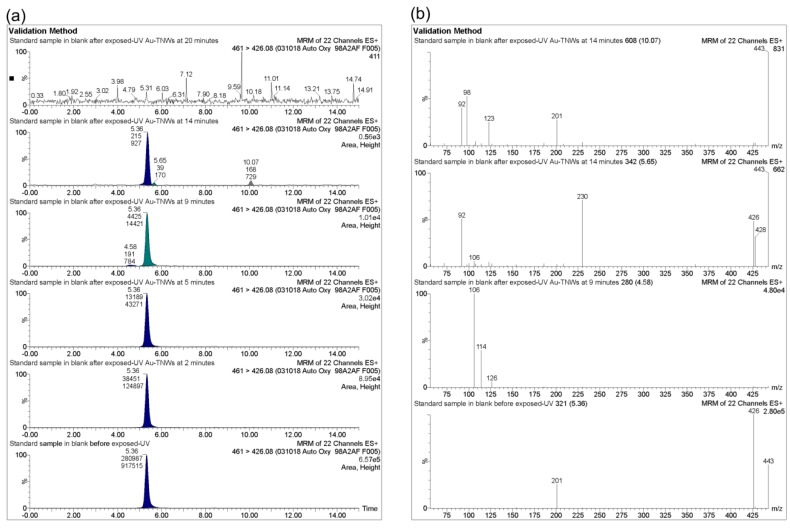
(**a**) Chromatogram ultra performance liquid chromatography (UPLC)-MS/MS photocatalytic degradation kinetic model of oxytetracycline (OTC, 500 ng/mL) with exposure to UV-VIS and Au-TNWs/TNAs. (**b**) Chromatogram UPLC-MS/MS impurities of OTC decomposition with exposed-UV-VIS and Au-TNWs/TNWs at reaction times of 9 and 14 min.

For the typical LC-MS/MS analysis in more detail, Figure 6a illustrated photocatalytic kinetic analysis of OTC at various reaction times of 0, 2, 5, 9, 14, and 20 min using Au-TNWs/TNAs and UV-VIS irradiation. As a result, removal percentage of OTC increased dramatically as a function of reaction time, and obtained 100% at 20 min. This indicates that antibiotics can completely degrade using the photocatalytic reaction with TiO_2_-based nanomaterials. Additionally, the UV-VIS photolysis or photocatalysis of antibiotics can produce potentially harmful substances [47,68]. Figure 6b shows the mass spectra of intermediates of OTC after 9 and 14 min of photocatalytic reaction. It is observed that intermediates separate at retention times of 4.58, 5.65, 10.97 min, respectively. At first, the OTC derived molecule 460.01 m/z is observed with a precursor ion [M-H]^+^ 461.01 in positive mode for the pristine blank sample. In monitoring reaction mode, there are only three product ions with the transition of m/z 461 → 426, 443 and 201 m/z. Meanwhile, after exposure to UV-VIS and Au-TNWs/TNAs, new product impurity ions with 126, 114, 126 m/z appeared at retention times of 4.58 min; ions 230, 106, 92 m/z at a retention time of 5.65 min, and 123.98, 92 m/z at 10.07 min also appeared. These results suggested the presence of decomposed products of the investigated antibiotics. 

## 4. Conclusions 

In this study, TiO_2_-based nanomaterials (i.e., TNAs, TNWs/TNAs, Au-TNAs, and Au-TNWs/TNAs) were developed toward the end of enhanced photocatalytic degradation of popular antibiotics. All the four kinds of nanomaterials exhibited the anatase phase with (004) and (101)-preferred orientation, grain size of 21.3–24.7 nm, and a similar crystallinity. The morphology of the samples was highly uniform and well-defined, which is promising for enhanced photocatalytic activity. In addition, we proposed and shed light on the formation mechanisms of TNAs and TNWs/TNAs. The nanomaterials were utilized for evaluating the photocatalytic degradation of antibiotics in model aquaculture wastewater by an LC-MS/MS method. The photocatalytic activity of TNWs/TNAs was higher than that of TNAs, primarily owing to the larger surface area of the former than the latter. By decorating Au NPs onto TNAs or TNWs/TNAs, the photocatalytic activity of Au-TNAs and Au-TNWs/TNAs was enhanced significantly compared to that of TNAs and TNWs/TNAs, because of the local surface plasmon resonance effect. Consequently, the Au-TNWs/TNAs achieved the highest activity for decomposition of antibiotics under UV-VIS or VIS irradiation. Based on the photocatalysis’s kinetic results, the photolysis of the eight antibiotics is of great concern. It was found that the photolysis of antibiotics depends on the stability of their structures. Indeed, the beta-lactam group (AMOX, AMPI) is more sensitive to photolysis than the sulfonamides group (SMT, SMZ) under UV-VIS irradiation. The photo-degradation pattern of more stable antibiotics (i.e., LCM, DXC, OTC, and VCM) followed pseudo-first order kinetics well, and their reaction rate constants were 0.26, 0.46, 0.54, and 0.51 min^−1^, respectively. Furthermore, the appearance of transformation products of the investigated antibiotics was evident after the chromatographic analyses, whose identification is of interest for future studies.
